# NVCL-Based Galacto-Functionalized and Thermosensitive Nanogels with GNRDs for Chemo/Photothermal-Therapy

**DOI:** 10.3390/pharmaceutics14030560

**Published:** 2022-03-03

**Authors:** Mirian A. González-Ayón, Jacob Licea-Rodriguez, Eugenio R. Méndez, Angel Licea-Claverie

**Affiliations:** 1 Centro de Graduados e Investigación en Química, Tecnológico Nacional de México/Instituto Tecnológico de Tijuana, Apartado Postal 1166, Tijuana 22454, Mexico; 2 División de Física Aplicada, Centro de Investigación Científica y Educación Superior de Ensenada, Carretera Ensenada-Tijuana No. 3918, Ensenada 22860, B. C., Mexico; jlicea@cicese.mx (J.L.-R.); emendez@cicese.edu.mx (E.R.M.); 3 Cátedras CONACYT-Centro de Investigación Científica y Educación Superior de Ensenada, Ensenada 22860, B. C., Mexico

**Keywords:** nanogels, gold nanorod, cisplatin, doxorubicin, chemo- and photothermal therapy

## Abstract

Dual-function nanogels (particle size from 98 to 224 nm) synthesized via surfactant-free emulsion polymerization (SFEP) were tested as smart carriers toward synergistic chemo- and photothermal therapy. Cisplatin (CDDP) or doxorubicin (DOX) and gold nanorods (GNRDs) were loaded into galacto-functionalized PNVCL-based nanogels, where the encapsulation efficiency for CDDP and DOX was around 64 and 52%, respectively. PNVCL-based nanogels were proven to be an efficient delivery vehicle under conditions that mimic the tumor site in vitro. The release of CDDP or DOX was slower at pH 7.4 and 37 °C than at tumor conditions of pH 6 and 40 °C. On the other hand, in the systems with GNRDs at pH 7.4 and 37 °C, the sample was irradiated with a 785 nm laser for 10 min every hour, obtaining that the release profiles were even higher than in the conditions that simulated a cancer tissue (without irradiation). Thus, the present study demonstrates the synergistic effect of chemo- and photothermal therapy as a promising dual function in the potential future use of PNVCL nanogels loaded with GNRDs and CDDP/DOX to achieve an enhanced chemo/phototherapy in vivo.

## 1. Introduction

Nanogels are cross-linked polymers of nanometric size in the form of a three-dimensional network that combine the advantages of nanotechnology with the hydrophilicity and flexibility of hydrogels. They are considered promising drug carriers due to their large surface area, porous structure, good biocompatibility, and relatively easy functionalization of their surface. Additionally, when they can respond to internal or external stimuli by changing their structural characteristics and hydrophilic-hydrophobic balance, they are classified as smart nanogels [1].

Currently, different nanogels with tailored properties have been developed with potential applications in the treatment of cancer [2]. Some of them have been synthesized in response to stimuli such as temperature, pH, or enzymes, mainly focused on passive targeting [3]; while others have been developed by modifying their surface with biological ligands, such as proteins, polysaccharides, peptides, and other small molecules, for both passive and active targeting through recognition of cancer cell receptors [4]. The potential application of these systems has been investigated in improving chemotherapy and radiotherapy. On the one hand, different anticancer drugs have been encapsulated with the aim of achieving site-specific chemotherapy [5]. In addition, they have also been studied as encapsulants of metallic nanoparticles, such as hollow gold nanoshells, gold nanorods (GNRDs), quantum dots, carbon nanotubes, and graphene nanosheets to induce localized hyperthermia in cancer therapy [6,7,8,9,10,11]. Specifically, GNRDs show structure-dependent optical properties, with a tunable photothermal response to light [12], typically showing two absorption bands in the UV-vis spectrum, one transversal at 520 nm, while the other longitudinal can be tuned within the visible or near-infrared (NIR) spectrum. According to the literature, with a single treatment of only chemotherapy or photothermal therapy, it is difficult to completely eradicate tumor cells due to the lack of drug targeting and the uneven distribution of heat at the tumor site. Therefore, in order to completely eradicate tumor cells, chemotherapy and photothermal therapy have been combined. Recently, some studies have shown that the combination of these strategies using GNRDs has resulted in synergistic anticancer efficacy from in vitro and in vivo models [13]. In addition, it has been recently shown that gold nanoparticles are not only physical dose enhancers (ionization) but also cause chemical and biological impacts on cells. The physical effect occurs by the generation of photoelectrons and Auger electrons, while the chemical/biological effects include oxidative stress and reactive oxygen species production, DNA damage and reduced repair, cell cycle arrest, and bystander effects. These synergistic effects highlight the great importance of targeting GNRDs in cancer treatment [14,15].

Among stimulus-sensitive drug carriers, heat-sensitive polymers, such as poly(*N*-vinylcaprolactam) (PNVCL), induce a phase transition of its cross-linked polymer in water with increasing temperature above a critical value (T_VPT_). This thermo-responsiveness plays a key role, given that it can be adjusted to the human body temperature by copolymerization with hydrophilic comonomers, such as poly(ethylene glycol) (PEG), which allows having control over drug release based on temperature changes in the body. Furthermore, PNVCL is a biocompatible polymer, and the possible hydrolysis of the amide group of the NVCL may produce a polymeric carboxylic acid, which is innocuous for biomedical applications [16,17]. Therefore, these smart materials offer new promising perspectives for the development of next-generation therapeutic agents [17]. According to previous reports, nanogels from poly(NVCL-VP-PEGMA) and poly(NVCL-2MBA-PEGMA) [18], fibrinogen-graft-poly(*N*-vinylcaprolactam) [19], poly(NVCL-*co*-methacrylic acid)-PEG [20], PNVCL-poly(acrylamidoglycolic acid) [21], and poly(6-O-vinyladipoyl-d-galactose-ss-*N*-vinylcaprolactam-ss-methacrylic acid) [22], have potential as pharmaceutical carriers of the well-known chemotherapeutic agents 5-fluoruracyl (5FU) or doxorubicin (DOX).

**Scheme 1:**
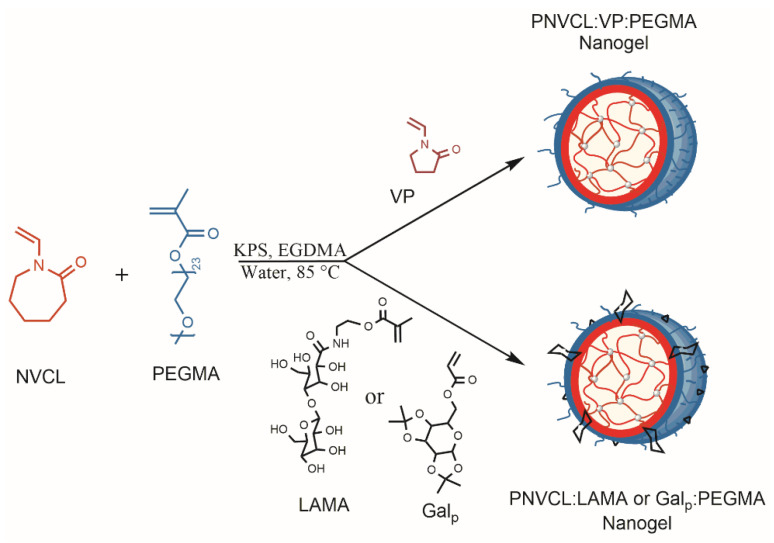
Core-shell nanogels synthesized by SFEP for encapsulation CDDP or DOX and GNRDs.

Previously, we developed two families of temperature-sensitive PNVCL-based nanogels via surfactant-free emulsion polymerization (SFEP) (Scheme 1). The first one, with tailored transition temperature (PNVCL:VP:PEGMA) [18], and the second one galacto-functionalized nanogels (PNVCL:GAL:PEGMA) [23], towards their potential use in the transport of anticancer drugs to target cancer cells, which could be driven by Galectin3-galactan affinity. In the present communication, we report the application of PNVCL-based nanogels as a dual-function system by loading a chemotherapeutic agent like cisplatin (CDDP) or DOX and a photothermal agent (GNRDs), as described below. The GNRDs encapsulated in the nanogels provide sensitivity to light [24] or electromagnetic radiation [25,26]. Small GNRDs can be heated via light or radiofrequency radiation. Irradiation with NIR light of the GNRDs on the PNVCL-based nanogels is expected to cause the nanogel to shrink, even when the GNRDs are bound to the surface of the nanogels [27]. CDDP and DOX were tested since those agents are widely used in chemotherapy while being very different: one organic molecule (DOX) and one metal-based inorganic compound. Additionally, the galactose linking onto the nanogels can provide the target-specific property by improving the interaction between the nanoparticles and specific receptors of tumor cells [28,29]. In this investigation, two types of experiments were carried out: first, CDDP or DOX were loaded into selected nanogels, and then in vitro release was evaluated under conditions that mimic a healthy (pH 7.4 and 37 °C) and cancerous (pH 6 and 40 °C) tissue. Subsequently, GNRDs were encapsulated in nanogels with CDDP or DOX, and the release of the drug was evaluated at pH 7.4 and 37 °C irradiated with a 785 nm laser for 10 min, every hour. These smart biomaterials are intended to improve cancer therapy, overcoming some drawbacks of conventional treatments.

## 2. Materials and Methods

### 2.1. Materials

*N*-Vinylcaprolactam (NVCL, 98%) was purified by recrystallization in hexane and dried under vacuum prior to use. Poly(ethylene glycol) methyl ether methacrylate (PEGMA, M_n_ = 950 gmol^−1^), and ethylene glycol dimethacrylate (EGDMA, 98%), were purified by filtering through an inhibitor remover column. The GAL monomer 2-lactobionamidoethyl methacrylate (LAMA) was synthesized following a procedure reported previously [23], while another commercially available GAL monomer was also used: 6-O-acryloyl-1,2,3,4-bis-O-(1-methyl-ethylidene)-α-d-galactopiranose (Gal_p_). *N*-vinylpyrrolidone (VP, 99%), potassium persulfate (KPS, 98%), *cis*-diammine-platinum (II) dichloride (CDDP), doxorubicin hydrochloride (DOX), cetyltrimethylammonium bromide (CTAB, 99%), gold (III) chloride hydrate, sodium borohydride (NaBH_4_, 99%), silver nitrate (AgNO_3_, 99%), sulfuric acid (H_2_SO_4_, 98%), and ascorbic acid (AA, 98%), were used as received. Deuterated and non-deuterated solvents and all reagents were purchased from Sigma-Aldrich (Toluca, Mexico); with one exception, commercial-grade distilled water (Sparkletts, Lakeside, CA, USA).

### 2.2. Preparation of NVCL-Based Nanogels

Nanogels were obtained by surfactant-free emulsion polymerization (SFEP) via a free radical mechanism, in accordance with previous reports [18,23]. In a typical experiment, the reaction mixture containing NVCL, PEGMA, EGDMA, and GAL-monomer in water, was added to a Schlenk flask containing a magnetic stir-bar and connected to a nitrogen inlet. The mixture was placed in a preheated oil bath at 85 °C, and after 20 min the thermal initiator, KPS, was added to the reaction system for starting the polymerization. The reaction was stopped after 30 min by cooling. Finally, the products were purified by dialysis (dialysis membrane Spectra/Por^®^, MWCO = 12–14 kDa) against deionized water for 5 days. Samples were frozen and lyophilized on a Labconco Freeze Dry System Freezone 4.5 (Kansas City, MI, USA). Then, the products were stored in a refrigerator until use. Particle size was determined by dynamic light scattering (DLS) using a Malvern Instruments Zetasizer Nano-ZS (ZEN 3690) (Malvern-Panalytical, Malvern, Worcestershire, UK) equipped with a red laser (630 nm) and a measurement detector at 90°. Using the same equipment, the response temperature (T_VPT_) of the nanogels was analyzed by DLS. The effect of temperature on the particle size was studied using dispersions at 1 mg mL^−1^ of nanogels in water, while the temperature was varied from 20 to 50 °C with 4 min of equilibration time between each temperature value. The reported T_VPT_ was taken as the minimum of the derivative of dD_h_/dT.

### 2.3. Loading of Drugs into Nanogels

CDDP and DOX were used as model anticancer drugs to evaluate the loading and release capacity of nanogels, using the equilibrium swelling method. For the encapsulation of CDDP or DOX, the first 10 mg of the drug were dispersed in 5 mL of deionized water, and then 20 mg of dry nanogels were dispersed in this solution, and the mixture was stirred for 24 h. Afterward, the drug-loaded nanogels were dialyzed against water for 4 h to remove surface adsorbed and non-encapsulated drug, using a membrane Spectra/Por^®^, MWCO: 12–14 kDa. The loaded nanogels were frozen at −4 °C in a conventional freezer for 12 h and then placed into the drying chamber of a Labconco Freeze Dry System Freezone 4.5 (Kansas City, MO, USA), precooled to −50 °C; drying was performed at 0.05 mbar for 24 h. The drug-loaded nanogels were dispersed in a PBS buffer at pH 6 or pH 7.4 and assayed spectroscopically using an ICP (Inductively Coupled Plasma) for CDDP content; while for DOX content, a UV-vis Cary 100 Spectrophotometer (Varian) at 480 nm was employed. The drug loading (%DL) and encapsulation efficiency (%EE) were calculated using Equations (1) and (2), respectively [20]:

(1) $$\%\mathrm{DL}=\left(\frac{\mathrm{MDng}}{\mathrm{mass}\;\mathrm{of}\;\mathrm{nanogels}\;+\mathrm{MDng}}\right)\times 100$$

(2) $$\%\mathrm{EE}=\left(\frac{\mathrm{initial}\;\mathrm{MD}-\mathrm{MD}\;\mathrm{supernant}}{\mathrm{initial}\;\mathrm{MD}}\right)\times 100$$

where MD is the mass of drug and MDng is the mass of drug in the nanogel.

### 2.4. Loading of GNRDs into Nanogels

Gold nanorods (GNRDs) were synthesized in an aqueous CTAB solution using a seed growth method [30]. Briefly, gold seeds were prepared by adding HAuCl_4_ (10 mM, 0.25 mL), to CTAB (0.1 M, 7.5 mL), then NaBH_4_ (10 mM, 600 μL) was added under stirring. Growth solution was prepared by adding HAuCl_4_ (10 mM, 5 mL) to CTAB (0.1 M, 100 mL) solution, and then AgNO_3_ (10 mM, 1 mL) and ascorbic acid (100 mM, 0.8 mL) were added. Next, the seed solution (7.5 mL) was added to a growth solution (0.25 mL). Centrifugation (11,000 rpm for 10 min) was used for purification. GNRDs were characterized by transition electron microscopy (TEM) and UV-vis spectroscopy according to procedures previously reported [30].

To load GNRDs into the nanogel, a GNRD solution (50 μg/100 μL deionized water) was added into a powdery state of nanogel (3 mg) using a vortex; this was followed by incubation at room temperature for 12 h, and then incubation at 4 °C for 12 h. The mixture solution remained motionless at room temperature for 24 h. Next, the liquid part, which contained the stabilized GNRDs in the nanogels, was carefully separated from the unstable GNRDs that precipitated in the container. The precipitate was placed in a pre-weighed vial that was subsequently placed in a vacuum oven at 40 °C for 48 h to dry completely and then reweighed. The percentage of GNRDs loaded was evaluated by weight difference between the original amount of GNRDs added to the solution and the weight of the precipitated GNRDs. The data obtained in percentage are shown in Table S2 (Supplementary Information file). The liquid part, GNRDs loaded in nanogels, were then frozen and lyophilized in a Labconco Freeze Dry System Freezone 4.5 (Kansas City, MO, USA). Then, the GNRD-loaded nanogels were stored in a freezer until use.

### 2.5. In Vitro Drug Release Profile of CDDP/DOX

In vitro release studies were performed under sink conditions at controlled temperature and agitation. Two operating conditions were established. On the one hand, 37 °C and pH 7.4 were selected as conditions that mimic the environment of a healthy body, and on the other hand, 40 °C and pH 6 were selected as conditions that mimic the tumor microenvironment. In both cases, constant stirring was maintained at 90 rpm. Typically, 5 mg of drug-loaded nanogels were dispersed in 3 mL of the corresponding buffer and then placed in a dialysis bag, which was subsequently immersed in 300 mL of the release medium at the desired temperature. At predetermined time intervals, an aliquot (3 mL) was withdrawn, and 3 mL of clean PBS was added to keep the volume of the release medium constant. The percentage of drug released (%DR) was calculated by Equation (3) [18]:

(3) $$\%\mathrm{DR}=\left(\frac{\mathrm{mass}\;\mathrm{of}\;\mathrm{drug}\;\mathrm{released}\;\mathrm{from}\;\mathrm{nanogels}}{\mathrm{mass}\;\mathrm{of}\;\mathrm{total}\;\mathrm{drug}\;\mathrm{loaded}\;\mathrm{in}\;\mathrm{nanogels}}\right)\times 100$$

Similar to the quantification of the loaded drug into nanogels, the evaluation of the drug released from them was performed spectroscopically by ICP (inductively coupled plasma) for CDDP and by UV-vis spectrophotometry for DOX.

## 3. Results

### 3.1. Preparation of PNVCL-Based Nanogels

**Figure 1:**
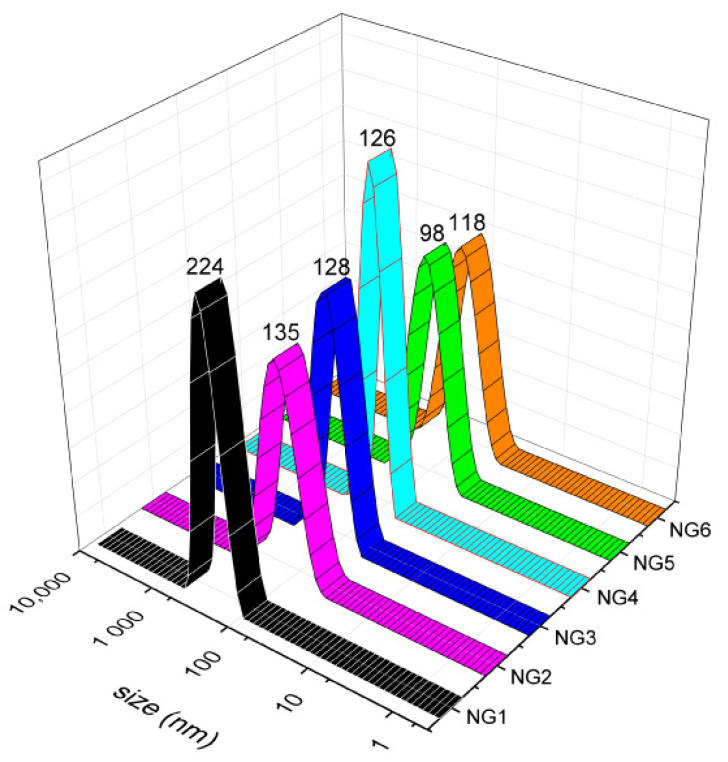
Size distribution by intensity of PNVCL-based nanogels containing VP or GAL.

The synthesis of thermoresponsive PNVCL-based nanogels was carried out via PEGMA-mediated SFEP. This method allows the preparation of core-shell nanogels with controlled size and responsive behavior, as previously reported. In this contribution, two families of nanogels were used: PNVCL:PEGMA nanogels with VP, synthesis described in [18] for tuning the transition temperature (samples NG1 to NG3 in Table 1), and PNVCL:PEGMA nanogels with galactose (GAL), described in [23] to target cancer cells (samples NG4 to NG6 in Table 1). In the case of nanogels with galactose, Gal_p_ or LAMA were used. As determined gravimetrically, conversions were between 50 and 60 wt% (Table 1). Colloidal stable particles were characterized by means of DLS, ^1^H-NMR, DSC, and other methods (see Table S1 and Figures S1–S10 in Supplementary Information File). The nomenclature used according to the molar composition is the following: PNVCL_92_:PEGMA_8_ (NG1), PNVCL_60_:VP_21_:PEGMA_19_ (NG2), PNVCL_53_:VP_26_:PEGMA_21_ (NG3), PNVCL_60_:GAL_p23_:PEGMA_17_ (NG4), PNVCL_68_:LAMA_6_:PEGMA_26_ (NG5), and PNVCL_81_:VP_8_:LAMA_3_:PEGMA_8_ (NG6). The size for all nanogels was obtained by DLS (Figure 1).

### 3.2. Drug Loading and In Vitro Release Study

**Figure 2:**
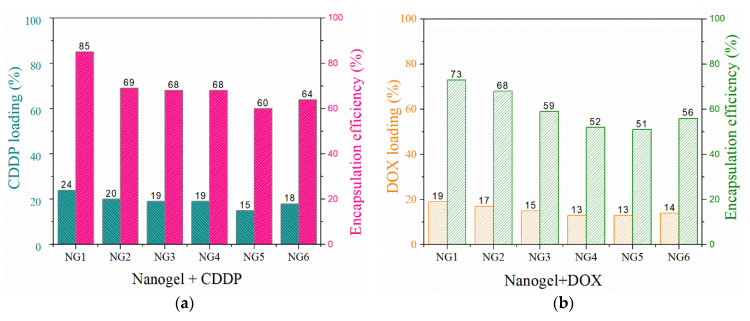
Drug loading and encapsulation efficiency of drugs in nanogels: (**a**) CDDP, (**b**) DOX.

The drug loading and release from nanogels depends on factors related to the polymer network (composition, structure, and size), the guest molecule (chemical structure and molecular weight), the possible chemical interactions that occur between both, and the release conditions (temperature, pH). In the case of CDDP and DOX, they are DNA inhibitor drugs widely used in chemotherapy [31,32], which have been encapsulated in nanogels and microgels [33,34]. In order to overcome the disadvantages of conventional administration of these drugs, the potential of nanogels based on PNVCL:VP:PEGMA and PNVCL:GAL:PEGMA for encapsulation and release of CDDP or DOX was tested in our study. Figure 2 shows the drug loading and encapsulation efficiency of CDDP (Figure 2a) or DOX (Figure 2b) into nanogels. For galacto-functionalized nanoparticles, the encapsulation efficiency for CDDP and DOX was, on average, 64 and 53%, respectively.

**Figure 3:**
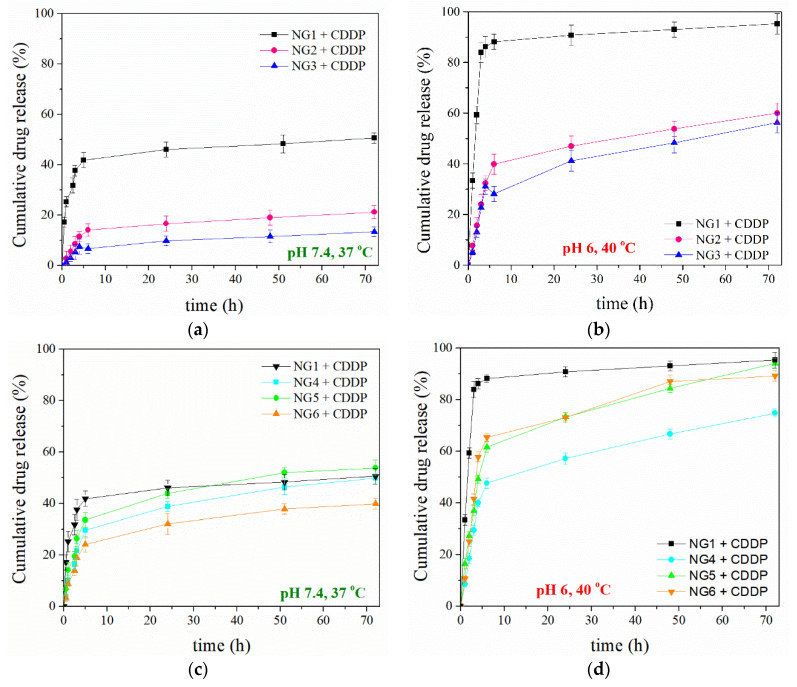
Cumulative CDDP release from nanogels at different conditions. From PNVCL:VP:PEGMA nanogels: (**a**) at pH 7.4, T = 37 °C; (**b**) at pH 6, T = 40 °C. From PNVCL:GAL:PEGMA nanogels: (**c**) at pH 7.4, T = 37 °C; (**d**) at pH 6, T = 40 °C.

**Figure 4:**
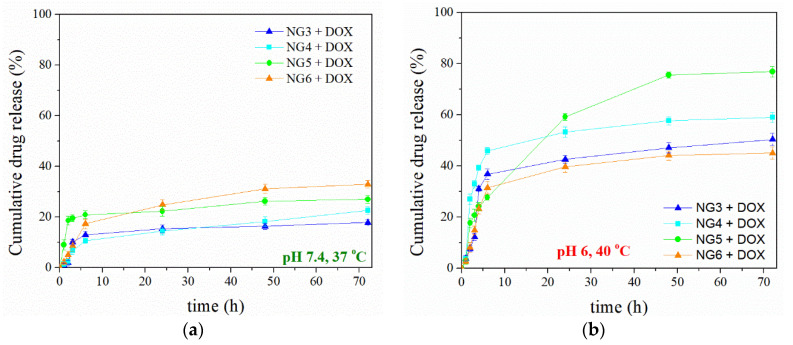
Cumulative DOX release from PNVCL:VP:PEGMA and PNVCL:GAL:PEGMA nanogels at (**a**) pH 7.4, T = 37 °C and at (**b**) pH 6, T = 40 °C.

According to previous studies, the normal pH of blood for sustaining human life is around 7.4, while tumor tissue has a lower extracellular pH, between 6.5 and 7.2, and drops to a pH from 5 to 5.5 when reaching the endosomes and lysosomes [35]. Therefore, in vitro drug release studies were carried out mimicking physiological conditions of healthy (pH 7.4 and 37 °C) and cancer (pH 6 and 40 °C) tissues. The pH was adjusted using 0.1 mM phosphate buffers. The nanogels were divided into two families: nanogels with a tailored transition temperature (PNVCL:VP:PEGMA) and galacto-functionalized nanogels (PNVCL:GAL:PEGMA). Figure 3a–d shows the cumulative release profiles for CDDP from these nanogels (NG1 to NG6), while Figure 4a,b display the release profiles of DOX using selected nanogels (NG3 to NG6). From these trials, it was demonstrated that there is a marked effect of temperature and pH on the cumulative drug release for the designed systems.

Mathematical models were applied to evaluate the kinetics of CDDP (Table 2) and DOX (Table 3) release from nanogels. Data were fitted by Equations (4)–(6) using a linear and no linear regression analysis [36].

(4) $$First\;order:\;F=1-e^{-kt}$$

(5) $$Higuchi\;equation:\;F=kt^{0.5}$$

(6) $$Peppas\;equation:\;F=kt^{n}$$

where: *F* is fractional drug release, *k* is release rate constant for the different equations, *n* is the diffusional exponent.

### 3.3. GNRDs Encapsulation and Photothermal Studies

**Figure 5:**
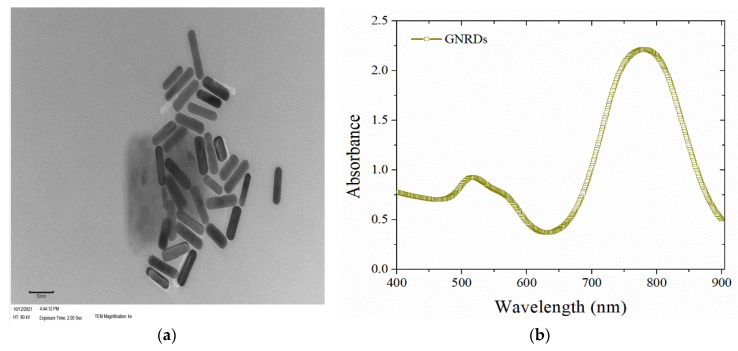
(**a**) TEM micrography of colloidal gold nanorods of aspect ratio of 3.75. (**b**) UV-vis spectrum showing the localized surface plasmon resonance of gold nanorods.

**Figure 6:**
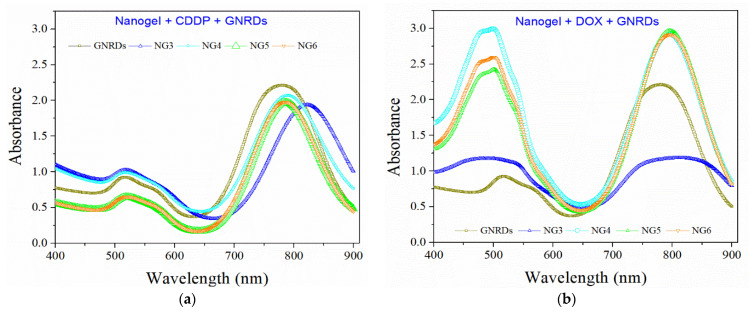
UV-vis spectra of GNRDs: (**a**) GNRDs/CDDP loaded in nanogels; and (**b**) GNRDs/DOX loaded in nanogels.

Gold nanorods were synthesized by the seed growth method. The product was characterized by transition electron microscopy (Figure 5a) and UV-vis spectroscopy (Figure 5b). Subsequently, GNRDs and CDDP or DOX were encapsulated in selected nanogels from PNVCL:VP:PEGMA, PNVCL:GAL:PEGMA, and PNVCL:VP:GAL:PEGMA, checking the success of the encapsulation by UV-vis spectroscopy (Figure 6). Nanogels NG3 to NG6 were selected for dual encapsulation of GNRDs-drug because they are the ones that showed smaller size, higher transition temperature, and because of their LAMA content, in addition to their relatively low percentage of CDDP or DOX release in systems that mimic healthy tissue conditions (without GNRDs).

**Figure 7:**
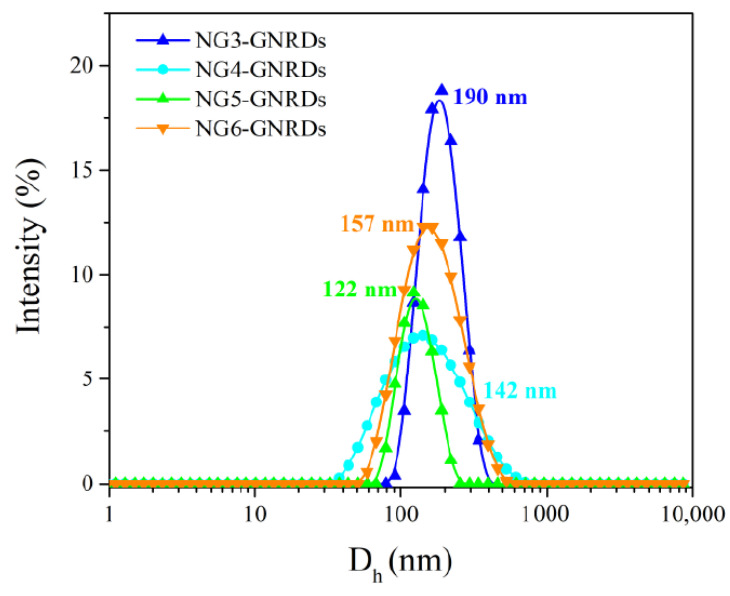
Size distribution by intensity of GNRDs encapsulated into PNVCL-based nanogels.

The encapsulation of GNRDs in nanogels was also verified via analysis of D_h_ by DLS (Figure 7). As expected, the results showed an increase in size after the incorporation of the metallic nanoparticles in the polymeric networks.

**Figure 8:**
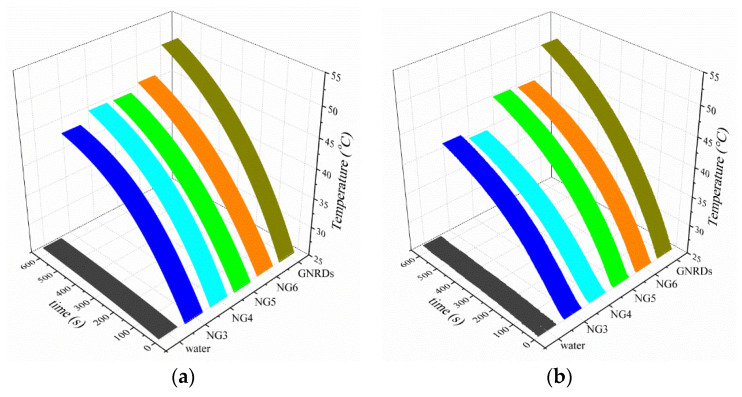
In situ photothermal effect monitoring of solution of: (**a**) GNRDs/CDDP-loaded nanogels, (**b**) GNRDs/DOX-loaded nanogels.

To confirm the GNRDs’ photothermal effect, a 785 nm laser was used to irradiate the samples (Figure 8). The measurement consisted of irradiating the sample for 10 min; during this time, the temperature data was recorded every 2 s. The efficacy of photothermal analysis is based on the intense electromagnetic fields associated with the metal surface, which convert the energy from absorbed radiation into heat, resulting in irreparable cell damage. For these measurements, first distilled water was irradiated, showing no increase in temperature. Then, the GNRDs dispersed in distilled water were irradiated, showing a huge temperature increase of 26 °C; that is, going from 25 to 51 °C. Likewise, 1 mg/mL dispersions of nanogels loaded with GNRDs were irradiated under the same conditions.

### 3.4. NIR-Irradiation Triggered CDDP and DOX Release

**Figure 9:**
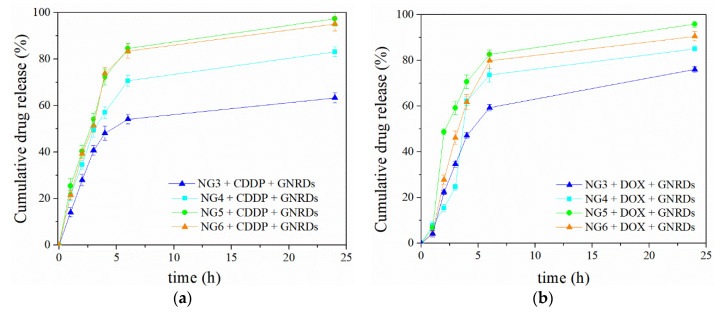
Cumulative drug release at pH 7.4 and 37 °C from PNVCL:VP:PEGMA and PNVCL:GAL:PEGMA nanogels with GNRDs. The samples were irradiated with an NIR laser (500 mW) for 10 min every hour until 6 h followed by a measurement after 24 h of total incubation time: (**a**) nanogel-CDDP-GNRDs and (**b**) nanogel-DOX-GNRDs.

Release experiments of CDDP or DOX were carried out from nanogels loaded with drug and GNRDs by intermittent irradiation of samples, with a 785 nm laser (CNI, MDL-III-785 nm-500 mW), for 10 min every hour, for the first 6 h followed by a last measurement at 24 h of total incubation time; the release profiles at healthy tissue conditions (pH 7.4 and 37 °C) are shown in Figure 9. It can be seen that there is a strong effect of the platform composition on the control of host molecule release, which was improved using galacto-functionalized nanogels.

## 4. Discussion

The aim of the present study was to evaluate the capacity of PNVCL-based nanogels to encapsulate anticancer drugs (CDDP or DOX) and metallic nanoparticles (GNRDs) for a future application in chemo- and photothermal therapy against cancer. The nanogels used for this study were characterized as previously reported [18,23], with the exception of nanogel NG6 synthesized for this study. Figure 1 shows the D_h_ distributions of nanogels with different contents of VP and GAL measured in water, where narrow unimodal distributions can be seen, with D_h_ between 98 and 224 nm and exhibiting polydispersity index (PDI) from 0.16 to 0.37; this demonstrates homogeneity of both types of nanogels (PNVCL:VP:PEGMA and PNVCL:GAL:PEGMA) in an aqueous medium. The content of NVCL, PEGMA, VP, and GAL in the respective nanogels was determined quantitatively by hydrogen nuclear magnetic resonance (^1^H-NMR) [18,23] and is shown in the Supplementary Information file. In general, data indicated a preferred incorporation of NVCL into nanogels with respect to the other monomers. On the other hand, the effect of temperature on the nanogel size was analyzed by DLS. Temperature-sensitive polymer networks are swollen below the T_VPT_ and shrink above it [37]. Regarding that, the effect of the comonomer concentration (VP or GAL) on the T_VPT_ was studied. NG1 exhibited a T_VPT_ close to 28 °C, while nanogels containing VP or GAL presented a T_VPT_ up to 42 °C. As expected, the incorporation of more hydrophilic monomers such as PEGMA or GAL in the nanogels increased the T_VPT_. Similarly, the incorporation of a monomer with a high transition temperature, such as *N*-vinylpyrrolidone (VP), also raised the response temperature of the materials obtained [38]. The response temperatures in phosphate buffers of pH 7.4 and 6 are similar to the values in pure water, showing differences of only one to two degrees.

The loading content of CDDP in nanogels NG1, NG2, NG3, NG4, NG5, and NG6 was between 15 and 24 wt%, whereas the encapsulation efficiency was within the range from 60 to 85 wt% (Figure 2a). Likewise, the content of DOX in nanogels was determined to be between 13 and 19%, while the encapsulation efficiency ranged from 51 to 73% (Figure 2b). In general, a slightly higher CDDP entrapment was registered, as compared with the DOX incorporation for the same type of nanogels. The encapsulation capacity using these drugs is attributed to the presence of hydrogen bonds and electrostatic forces between CDDP or DOX and the polymeric network, involving the amide of NVCL or VP and the ester group of PEGMA. In the case of galacto-functionalized nanogels, interactions can take place through the amide group and ester group of LAMA. Besides, the probable CDDP-polymer complexation could provide a secondary interaction between this drug and the nanogels, which also allows for controlling the burst-effect of the drug release depending on the pH and temperature [39]. In addition, the low molecular weight of CDDP, with respect to DOX, could favor better internalization of the drug inside the polymer network. Figure 3a,b shows the CDDP release profile from PNVCL:PEGMA (NG1) and PNVCL:VP:PEGMA (NG2 and NG3) at pH 7.4, 37 °C and pH 6, 40 °C, respectively. Meanwhile, Figure 3c,d shows the release profile of CDDP from galacto-functionalized nanogels NG4, NG5, and NG6, keeping NG1 as a reference. It can be observed that the drug release profiles depend on the nanogel composition and evidently are also influenced by the temperature and pH of the medium. Drug release from PNVCL:PEGMA, PNVCL:VP:PEGMA, and PNVCL:GAL:PEGMA is faster under conditions that mimic a cancer tissue as compared to healthy tissue; the CDDP release results in being slower from nanogels with higher T_VPT_. As an example, in nanogels with VP (NG2 and NG3), the T_VPT_ was 34 and 42 °C, respectively (Table 1), and the release profiles in both cases, simulating a healthy and cancer tissue (Figure 3a,b), were higher for the nanogel with lower T_VPT_ (NG2). Similarly, in galacto-functionalized nanogels, the T_VPT_ for NG5 and NG6 was 32 and 37.5 °C, respectively (Table 1). Drug diffusion from platforms with higher T_VPT_ than the assay temperature could be slower because the polymer network was not shrunken. To the opposite, drug diffusion was accelerated when the assay temperature was higher than the response temperature. This highlights the control that can be achieved with these engineered thermo-sensitive materials. For instance, from the CDDP release profile at conditions that mimic a healthy tissue (T = 37 °C), the release from NG5 (T_VPT =_ 32 °C) was considerably higher than from NG6 (T_VPT_ = 37.5 °C), showing a difference of around 20% of cumulative release of the drug. However, at conditions that mimic a cancer tissue (T = 40 °C), there were no significant differences between both systems, NG5 and NG6 reached around 90% as maximum release at 72 h, since, in the last case, the assay temperature was higher than the response temperature of both nanogels.

Considering the systems with the lowest percentage of CDDP released under healthy cell conditions, nanogels NG3, NG4, NG5, and NG6 were selected to load and release another model drug, doxorubicin. The cumulative DOX released up to 72 h was around 20%, resulting in small increments from the NG3 to NG6 (Figure 4a). Meanwhile, under conditions that mimic a cancer tissue, the profiles show a considerable higher release, reaching up to 70% of accumulative drug release in the case of nanogel NG5 (with lower T_VPT_ than the assay temperature) (Figure 4b).

The results of fitting experimental data to mathematical models are presented in Table 2 for the release of CDDP from nanogels. The most appropriate model for the adjustment was selected according to linearity (*r*^2^). The best fitting for CDDP release corresponds to First-order and Peppas’s models. Values of *n* obtained by Peppas’s model can be used to judge the drug release mechanism and the matrix geometry [40]; if the *n* value is lower than 0.43, the kinetics of drug release from nanogels corresponds to a spherical matrix and a drug release mechanism controlled by Fickian diffusion. When it comes to drug release through a porous material, the value of *n* results to be less than 0.5 due to the combination of partial diffusion mechanisms through a swollen matrix and through water-filled pores. In addition, these values denote the existence of another simultaneous diffusion process; in this case, it is attributed to the shrinkage of the networks due to the action of temperature. The same analysis was made for the DOX release profiles that mimic healthy and cancer tissue conditions (Table 3). The best-fitting models, like in CDDP, were with the first order and Peppa’s models. In both cases, the release of CDDP or DOX from nanogels was controlled by Fickian diffusion. The values for the release rate constant (*k*), for both drugs, in all models were higher for the systems that mimic cancer tissue conditions compared to those that mimic healthy tissue, which confirms the potential of PNVCL:PEGMA, PNVCL:VP:PEGMA, and PNVCL:GAL:PEGMA as carriers of anticancer drugs into tumor tissues.

On the other hand, gold nanostructures have been studied as therapeutic agents designed to selectively destroy cancer cells by laser-induced photothermal heating without affecting healthy cells. Moreover, nanorods may find application in biomedical imaging, drug delivery, and photothermal therapy due to the localized surface plasmon resonance (LSPR) ranging from the visible to near-infrared (NIR) region. GNRDs were synthesized by a seed-mediated growth method. Figure 5a shows a transmittance electron microscope image; the results showed formation of gold nanorods (GNRDs) with a near-uniform length of 64 ± 4 nm and diameter of 14 ± 4 nm (3.75 aspect ratio). Figure 5b displays the observed optical absorption of GNRDs measured by spectroscopy UV-vis. GNRDs exhibited a strong long-wavelength band due to the longitudinal oscillation of electrons at 780 nm and a weak short-wavelength band around 520 nm due to the transversal electronic oscillation. The aspect ratio of 3.75 of GNRDs supports a possible application in photothermal therapy against cancer due to their absorption overlapping with a region of minimum extinction of the human tissues. These results agree with the reports from Murphy et al. [41]. The absorption band of the GNRDs also overlaps the laser wavelength at 785 nm that was used in the laboratory to irradiate samples.

The synthesized GNRDs were encapsulated on selected nanogels, observing, in all cases, a shift of the longitudinal LSPR to higher wavelengths (Figure 6). In the case of GNRDs with an LSPR longitudinal band of 767 nm, right after the purification, it was observed that the LSPR band was displaced to 780 nm. This phenomenon is attributed to the possible agglomeration of the gold nanoparticles without CTAB and a possible increase of the local refractive index [41]. For the encapsulated GNRDs, the LSPR longitudinal bands were further displaced to values around 818 nm (Figure 6a) and 795 nm (Figure 6b) for nanogels with CDDP and DOX, respectively. The content of GNRDs in the nanogels was determined by gravimetry; the results were around 15% by weight (see Table S2 in Supplementary Information file). The increase in absorbance at 480 nm in Figure 6b corresponds to the absorbance of DOX.

GNRD encapsulation in nanogels resulted in an increase in particle size. Figure 1 shows the sizes distribution by DLS for the empty nanogels, while Figure 7 gives the sizes of nanogels after GNRD encapsulation. The increases of D_h_ were 48, 13, 25, and 33% for NG3, NG4, NG5, and NG6, respectively. This increment suggested that the introduction of GNRDs into the nanogels impacted the cross-linked chains of the nucleus to be more extended and/or that a part of the metallic nanoparticles become trapped in the PEGMA shell. The results in Figure 8 show the thermal heating of the surroundings of the NG3, NG4, NG5, and NG6 nanogels in water when GNRDs were encapsulated. Figure 8a shows increases in temperature of 19, 21, 21, and 22 °C for nanogels NG3, NG4, NG5, and NG6, respectively, when CDDP and GNRDs were encapsulated. Meanwhile, in nanogels with DOX/GNRDs, the increases in temperature were 17, 16, 21, and 21 °C for the same materials, respectively (Figure 8b). In this sense, these materials have great potential to be used in photothermal therapy against cancer.

Studies of drug release mediated by NIR radiation were conducted. From the temperature increment studies, it was confirmed that the temperature reached, after 10 min of irradiation with a laser output power of 500 mW, was high enough to allow for a thermo-sensitive response of the nanogel, while the control of pure water did not show a noticeable temperature increase. The temperature-driven contraction of the nanogel may result in squeezing out the encapsulated drug (CDDP or DOX). The nanogels possessing high T_VPT_ values may minimize drug leakage during body circulation and release the drug when necessary since the incorporation of GNRDs in nanogels acts as “NIR absorbers”, allowing heating and resulting in drug release by laser application without damage to the neighbor tissue without GNRDs. Figure 9 shows the characteristics of the CDDP and DOX release from nanogels with irradiation by NIR light. The samples were irradiated for a period of 10 min, every hour, with a total time of 6 h. A faster release was observed upon NIR irradiation. After the first NIR exposure for 10 min, the percentage of CDDP release increased around 10%. When the samples were exposed to NIR light five times for 10 min, the accumulated released amount of CDDP over the whole experiment process was about four-fold greater than that without laser irradiation at pH 7.4 and 37 °C (Figure 9a). On the other hand, DOX released after the first irradiation increased to around 5%, and after five irradiations, the accumulated release increased to around 20% compared to the release without irradiation, reaching a cumulative release from 70% to near 100% (Figure 9b). In general, it was observed that the rate of drug release was controlled by the T_VPT_ of the nanogels, as compared to the assay temperature, and also a slightly acidic pH (6) accelerated the drug released due to the tertiary amine groups of the NVCL as a major component of the nanogels.

## 5. Conclusions

In summary, galacto-functionalized PNVCL-based nanogels were prepared as a multifunctional platform to combine passive and active drug delivery, NIR light-activated drug delivery, and photothermal therapy in one system simultaneously. The nanogels were obtained by surfactant-free emulsion polymerization with unimodal size distributions between 98 and 224 nm in hydrodynamic diameter. On the other hand, gold nanorods with a good aspect ratio were obtained. The resulting GNRDs, even after being encapsulated in the nanogels, showed surface plasmon absorbance within the NIR region, which allowed an NIR-induced temperature rise. Additionally, two model anticancer drugs (cisplatin and doxorubicin) were individually encapsulated in nanogels, obtaining loading percentages between 13 and 24% of each drug, favored by electrostatic interactions and hydrogen bonds between the drug and the polymer network. The drug release profiles indicated a low extent of release of CDDP and DOX from nanogels at conditions that mimic healthy tissue (pH 7.4 and 37 °C), and a high extent of release from the same nanogels that mimic cancer tissue (pH 6 and 40 °C), which was confirmed from the release rate constant (*k*) obtained by fitting experimental data to mathematical models. Peppas model suggested a Fickian diffusion of CDDP and DOX through spherical nanogels. In addition, the dual encapsulation of drug-GNRDs was performed, yielding Nanogel-CDDP-GNRDs and Nanogel-DOX-GNRDs and obtaining quite significant increases in the accumulated release in the systems with irradiation. The double encapsulation capacity of nanogels (drug + GNRDs) and the in vitro release profiles demonstrated the potential of PNVCL-based nanogels as drug delivery systems for chemo- and photothermal therapy against cancer.

## Figures and Tables

**Table 1:** Composition of nanogels, transition temperature, and yield.

Nanogel	Product Composition (mol%) ^a^				T_VPT_ (°C) ^b^			Yield (wt%)
	NVCL	PEGMA	GAL	VP	Water	pH 7.4	pH 6	
NG1	92	8	0	0	28	29	29	50
NG2	60	19	0	21	34	34	32	55
NG3	53	21	0	26	42	40	40	53
NG4	60	17	Gal_p_23	0	41	39	39	62
NG5	68	26	LAMA6	0	32	32	31	60
NG6	81	8	LAMA3	8	37.5	35	37	58

^a^ As determined by ^1^H-NMR; ^b^ as determined by DLS.

**Table 2:** Release rate for CDDP: in vitro release data fitted with different models.

Nanogel	Conditions for Release ^a^	First Order (*k*; *r*^2^) ^b^	Higuchi (*k*; *r*^2^) ^c^	Peppas (*k; n; r*^2^) ^d^
NG1	Healthy	0.014; 0.99	0.207; 0.98	0.235; **0.39**; 0.97
	Cancer	0.666; 0.98	0.516; 0.98	0.818; **0.03**; 0.99
NG2	Healthy	0.026; 0.98	0.031; 0.99	0.097; **0.29**; 0.99
	Cancer	0.088; 0.99	0.081; 0.99	0.747; **0.29**; 0.99
NG3	Healthy	0.020; 0.99	0.062; 0.99	0.042; **0.33**; 0.99
	Cancer	0.095; 0.98	0.262; 0.99	0.175; **0.34**; 0.99
NG4	Healthy	0.061; 0.99	0.123; 0.90	0.187; **0.38**; 0.98
	Cancer	0.114; 0.98	0.195; 0.93	0.248; **0.33**; 0.98
NG5	Healthy	0.070; 0.99	0.141; 0.94	0.170; **0.36**; 0.98
	Cancer	0.160; 0.99	0.255; 0.97	0.313; **0.36**; 0.99
NG6	Healthy	0.049; 0.99	0.118; 0.93	0.114; **0.39**; 0.98
	Cancer	0.192; 0.97	0.277; 0.95	0.325; **0.37**; 0.97

^a^ Mimic healthy (pH 7.4 and 37 °C) and cancer (pH 6 and 40 °C) tissue conditions. ^b^ Obtained by Equation (4). ^c^ Obtained by Equation (5). ^d^ Obtained by Equation (6).

**Table 3:** Release rate for DOX: in vitro release data fitted with different models.

Nanogel	Conditions for Release ^a^	First Order (*k*; *r*^2^) ^b^	Higuchi (*k*; *r*^2^) ^c^	Peppas (*k; n; r*^2^) ^d^
NG3	Healthy	0.026; 0.86	0.078; 0.99	0.114; **0.17**; 0.98
	Cancer	0.043; 0.99	0.166; 0.91	0.208; **0.36**; 0.98
NG4	Healthy	0.019; 0.96	0.056; 0.88	0.081; **0.37**; 0.93
	Cancer	0.107; 0.92	0.208; 0.98	0.389; **0.17**; 0.94
NG5	Healthy	0.087; 0.91	0.054; 0.87	0.185; **0.10**; 0.99
	Cancer	0.176; 0.92	0.215; 0.88	0.196; **0.41**; 0.90
NG6	Healthy	0.032; 0.99	0.069; 0.86	0.122; **0.39**; 0.99
	Cancer	0.065; 0.98	0.126; 0.83	0.218; **0.30**; 0.99

^a^ Mimic healthy (pH 7.4 and 37 °C) and cancer (pH 6 and 40 °C) tissue conditions. ^b^ Obtained by Equation (4). ^c^ Obtained by Equation (5). ^d^ Obtained by Equation (6).

## Data Availability

Not applicable.

## Supplementary Materials

The following supporting information can be downloaded at: https://www.mdpi.com/article/10.3390/pharmaceutics14030560/s1, Figure S1: Berry plot by SLS of nanogel NG2, Table S1. Characterization of nanogels, Table S2. GNRDs loading percentage in nanogels, Figure S2. Hydrodynamic diameter of nanogel NG1 as function of temperature in PBS at pH 7.4 and 6, Figure S3. Hydrodynamic diameter of nanogel NG2 as function of temperature in PBS at pH 7.4 and 6, Figure S4. Hydrodynamic diameter of nanogel NG3 as function of temperature in PBS at pH 7.4 and 6, Figure S5. Hydrodynamic diameter of nanogel NG4 as function of temperature in PBS at pH 7.4 and 6, Figure S6. Hydrodynamic diameter of nanogel NG5 as function of temperature in PBS at pH 7.4 and 6, Figure S7. Hydrodynamic diameter of nanogel NG6 as function of temperature in water and in PBS at pH 7.4 and 6, Figure S8. 1H-NMR spectrum of nanogel NG6, Figure S9. Thermogram by DSC of nanogel NG6, Figure S10. Thermogram by TGA of nanogel NG6.
