# Supplementary material for: NVCL-Based Galacto-Functionalized and Thermosensitive Nanogels with GNRDs for Chemo/Photothermal-Therapy
Source: Pharmaceutics. 2022 Mar 3;14(3):560. doi: 10.3390/pharmaceutics14030560 (PMC8951641; doi:10.3390/pharmaceutics14030560)
Supplement: Supplementary file 1 [file pharmaceutics-14-00560-s001.zip › pharmaceutics-1595012-supplementary.pdf]

pharmaceutics

MDPI

Supplementary material: NVCL-based galacto-functionalized and thermosensitive nanogels with GNRDs for chemo/photo-thermal-therapy

Mirian A. González-Ayón, Jacob Licea-Rodriguez, Eugenio R. Méndez and Angel Licea-Claverie

Metodology for determination of the content of GNRDs in nanogels

To load GNRDs into the nanogel, a GNRDs solution (50 g/100 L deionized water) was added into a powdery state of nanogel (3 mg) followed by incubation at room temperature for 12 h, and then incubate at 4 °C for 12 h. The mixture solution remained motionless at room temperature for 24 h. Next, the liquid part, which contained the stabilized GNRDs in the nanogels, was carefully separated from the unstabilized GNRDs that precipitated in the container. The precipitate was placed in a pre-weighed vial that was subsequently placed in a vacuum oven at 40 °C for 48 hours to dry completely and then reweighed. The percentage of GNRDs loaded was evaluated by weight difference between the original amount and the weight of the precipitated GNRDs. The data obtained in percentage are shown in **Table S2**. The liquid part, GNRDs loading into nanogels, were were frozen and lyophilized in a Labconco Freeze Dry System Freezone 4.5 (Kansas City, MI, USA). Then, the loading nanogels were stored in a freezer until use.

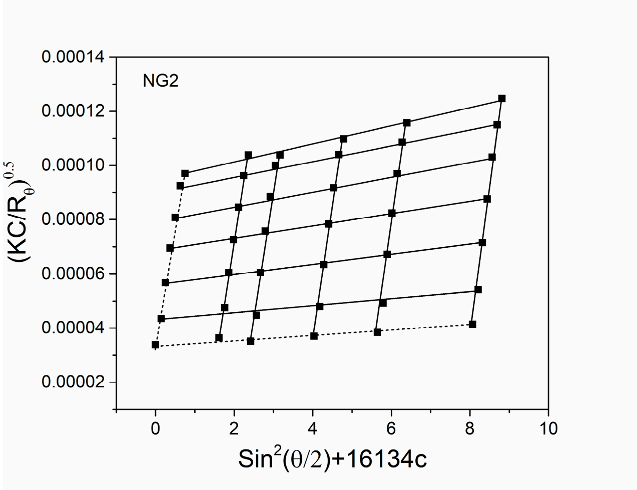

Figure S1. Berry plot by SLS of nanogel NG2.

Table S1. Characterization of nanogels

Nanogel	M_w [a] (g mol^{-1})	R_g [a] (nm)	A_2 [a] (mol mL g^{-2})	Zeta potential at pH 7.4(mV)	Reference
NG1	1.11×10^8	143	2.64×10^{-6}	−10	1, 2
NG2	2.2×10^8	70	3.75×10^{-7}	-	-
NG3	4.67×10^7	98	1.86×10^{-6}	-	1
NG4	8.74×10^8	76	4.83×10^{-7}	−12	2
NG5	3.53×10^6	43	4.01×10^{-6}	−25	2
NG6	-	-	-	−21	-

[a] Data obtained by SLS in water at 25 °C.

Table S2. GNRDs loading percentage in nanogels.

Nanogel	GNRDs loading (wt%)
NG1	14
NG2	15
NG3	14
NG4	16
NG5	16
NG6	15

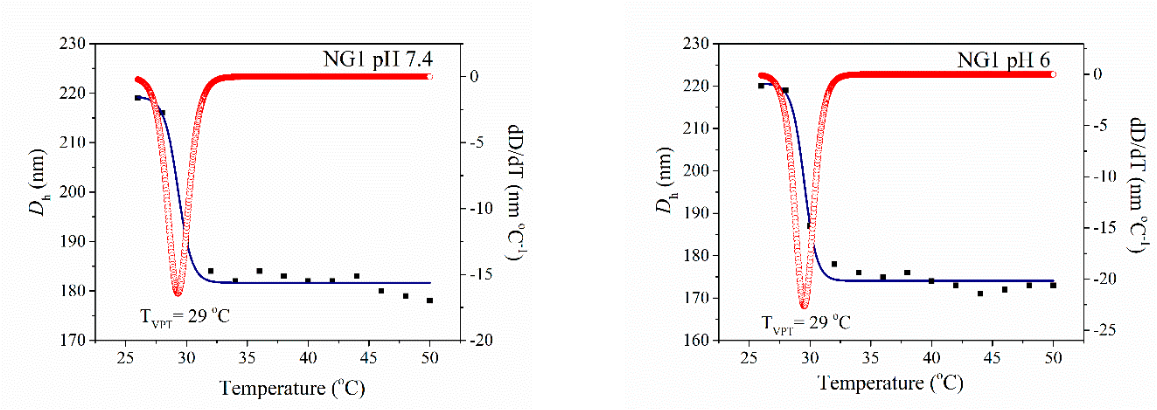

Figure S2. Hydrodynamic diameter of nanogel NG1 as function of temperature in PBS at pH 7.4 and 6.

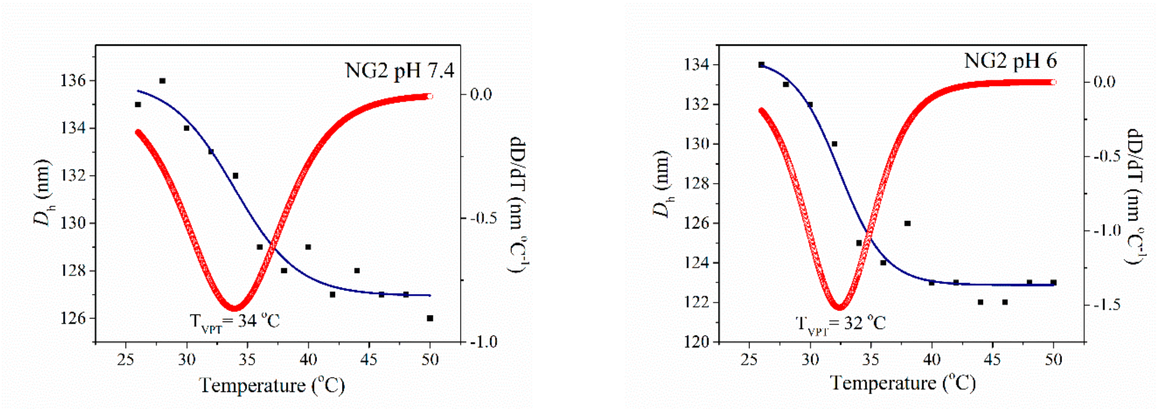

Figure S3. Hydrodynamic diameter of nanogel NG2 as function of temperature in PBS at pH 7.4 and 6.

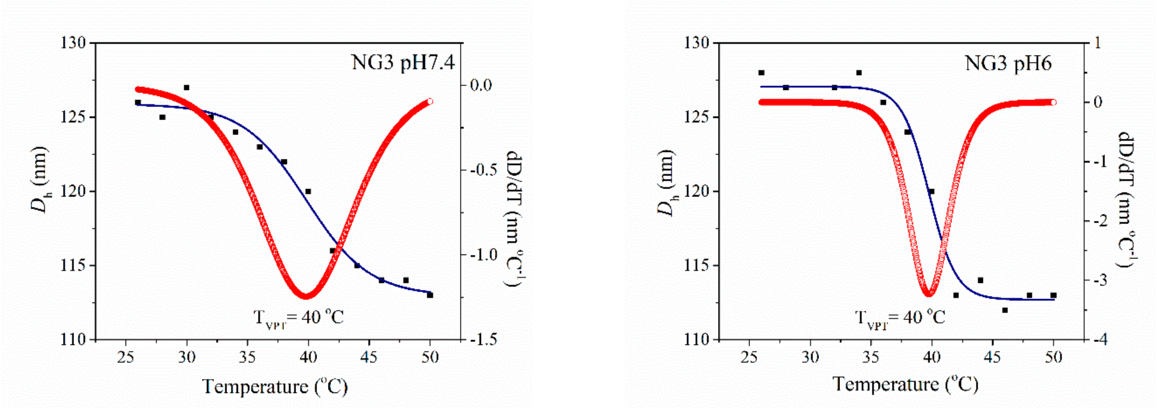

Figure S4. Hydrodynamic diameter of nanogel NG3 as function of temperature in PBS at pH 7.4 and 6.

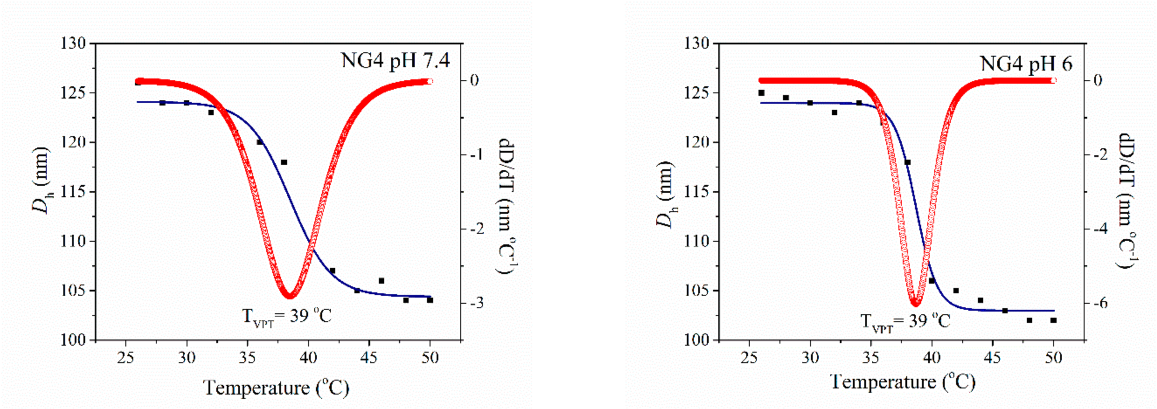

Figure S5. Hydrodynamic diameter of nanogel NG4 as function of temperature in PBS at pH 7.4 and 6.

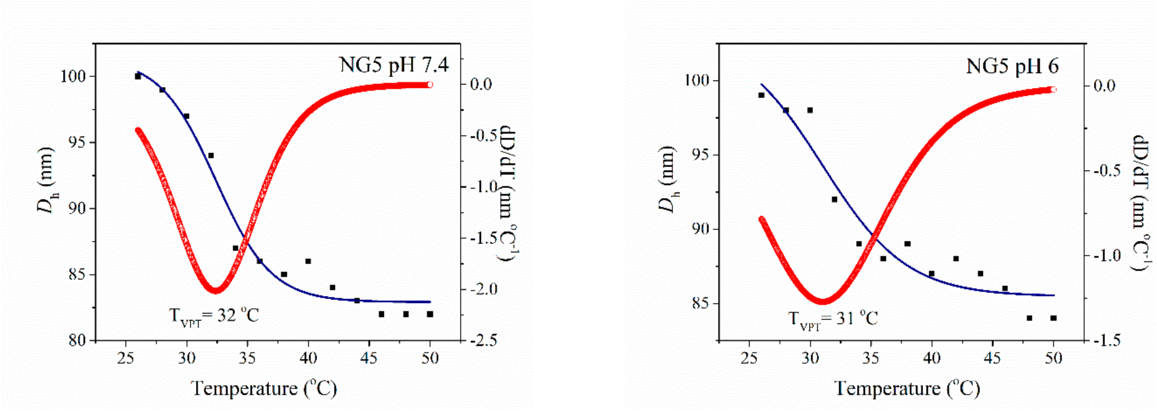

Figure S6. Hydrodynamic diameter of nanogel NG5 as function of temperature in PBS at pH 7.4 and 6.

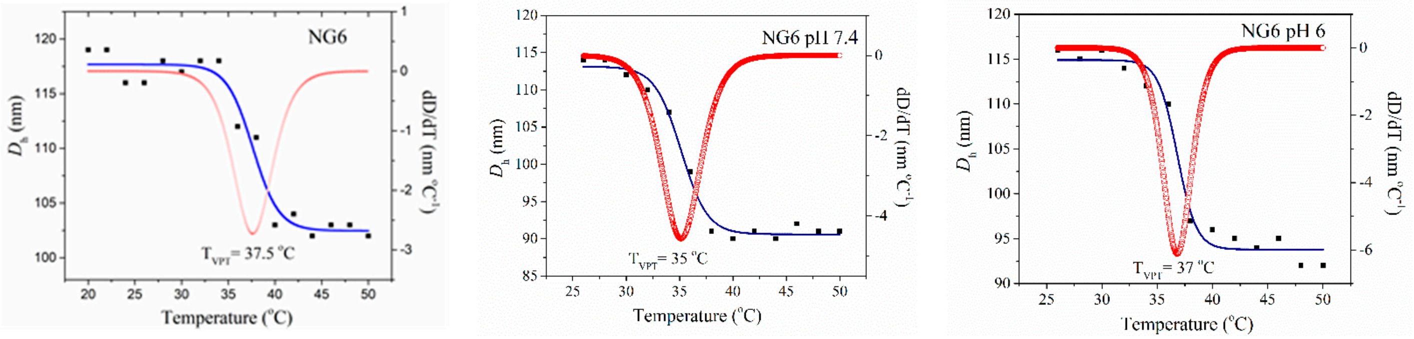

Figure S7. Hydrodynamic diameter of nanogel NG6 as function of temperature in water and in PBS at pH 7.4 and 6.

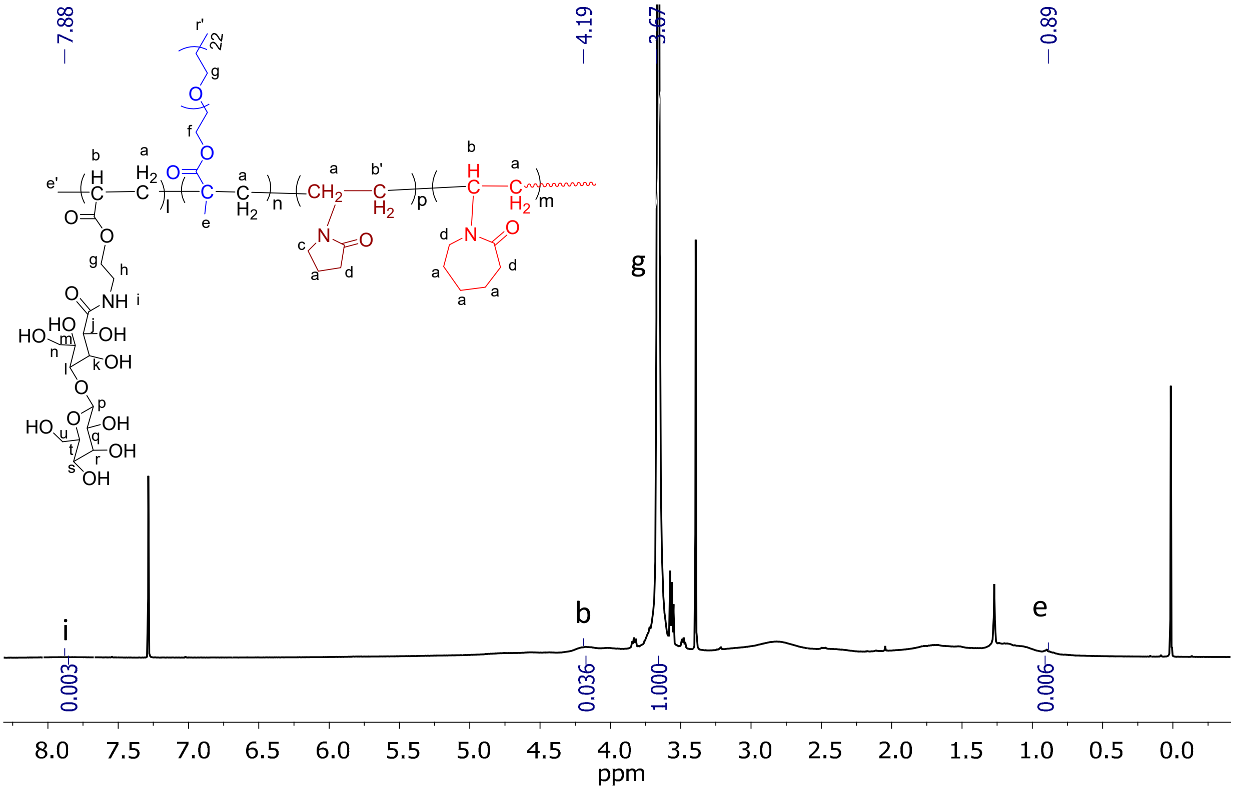

Figure S8. ^{1}H-NMR spectrum of nanogel NG6.

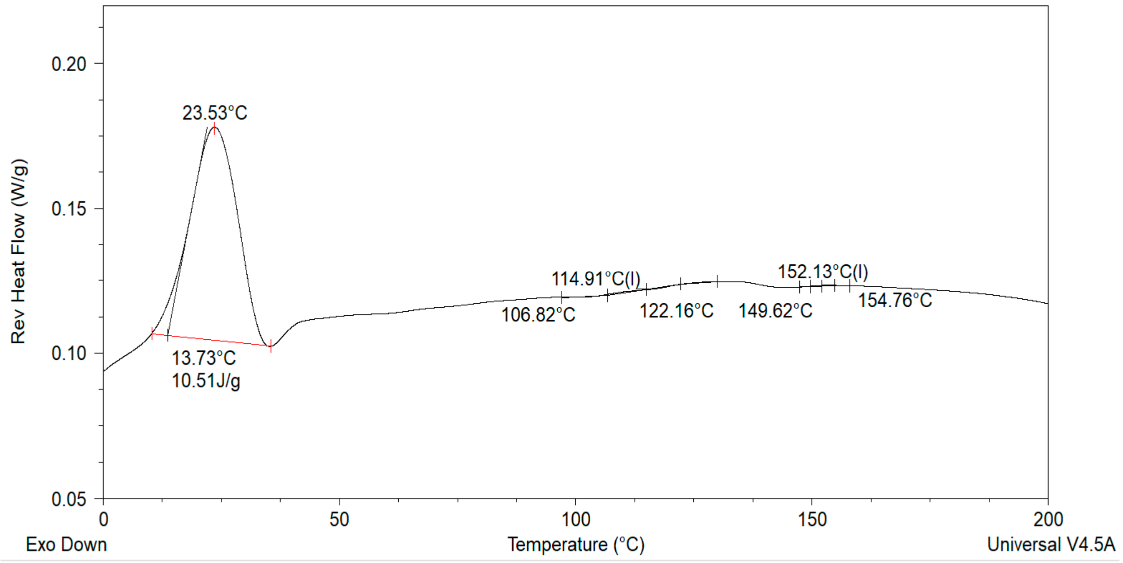

Figure S9. Thermogram by DSC of nanogel NG6.

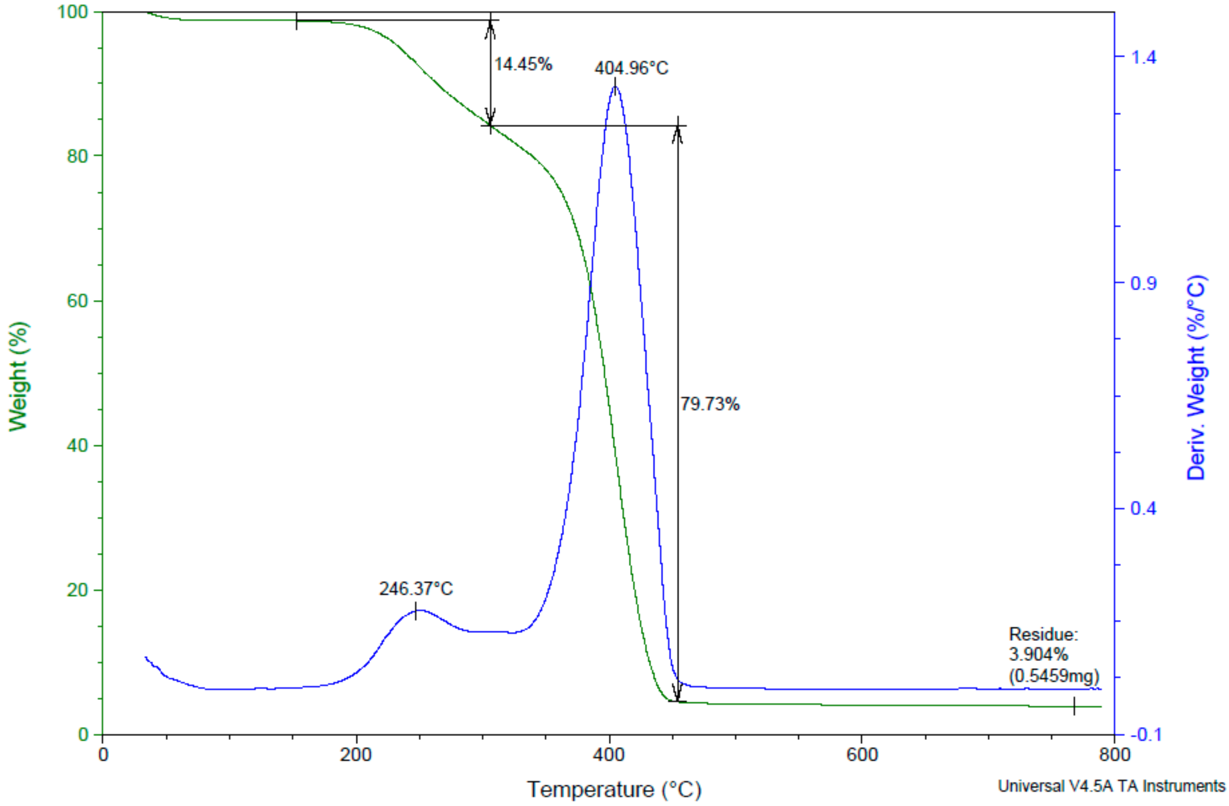

Figure S10. Thermogram by TGA of nanogel NG6.

References

1. González-Ayón, M. A.; Sañudo-Barajas, J. A.; Picos-Corrales, L. A.; Licea-Claverie, A. PNVCL-PEGMA nanohydrogels with tailored transition temperature for controlled delivery of 5-fluorouracil. *J Polym Sci A Polym Chem* **2015**, *53,* 2662-2672.
2. González-Ayón, M. A.; Licea-Claverie, A.; Sañudo-Barajas, J. A. Different strategies for the preparation of galactose-functionalized thermos-responsive nanogels with potential as smart drug delivery systems. *Polymers* **2020**, *12,* 2150.
